# The impact of a pay-for-performance system on timing to hip fracture surgery: experience from the Lazio Region (Italy)

**DOI:** 10.1186/1472-6963-13-393

**Published:** 2013-10-07

**Authors:** Paola Colais, Luigi Pinnarelli, Danilo Fusco, Marina Davoli, Mario Braga, Carlo A Perucci

**Affiliations:** 1 Department of Epidemiology, Regional Health Service, Via Santa Costanza 53, Rome, Lazio Region 00198, Italy; 2 Department of Social and Economic Planning, Rome, Lazio Region, Italy; 3 National Agency of Regional Health Services, Rome, Italy

**Keywords:** Pay-for-performance, Hip fracture, Surgery, Information systems

## Abstract

**Background:**

A tariff modulation mechanisms has been introduced in some Italian regions with the aim of reducing inappropriate admissions and improving quality of care. In response to a regional act, hospitals in Lazio adopted a clinical pathway for elderly patients with hip fracture and introduced a compensation system based on the quality of health care, as in a pay-for-performance model. The objective of the present study was to compare the proportion of surgery for hip fracture performed within 48 hours of admission among Lazio hospitals according to different payment systems, before and after the implementation of the regional act.

**Methods:**

A retrospective cohort study of patients aged 65 years and over, residing in the Lazio region and admitted to an acute care hospital for hip fracture before (1 July 2008 - 30 June 2009) and after (1 July 2010 - 30 June 2011) the pay-for-performance act. The proportion of surgeries performed within 48 h of hospital arrival was calculated. An adjusted multivariate regression analysis was applied to assess the effect of hospital payment type on the likelihood of surgery within 48 h of hospital arrival.

**Results:**

The share of patients with hip fracture that had surgery within 48 hours was 11.7% before the introduction of the pay-for-performance act and 22.2% after. The proportion of early hip fracture operations increased after the pay-for-performance act, regardless of hospital payment type. The largest increase of surgery within 48 h occurred in private hospitals (adjusted Relative Risk = 2.80, p < 0.001).

**Conclusions:**

The introduction of a compensation system based on health care quality is associated with improved quality of care for elderly patients with hip fracture, especially in hospitals that only use the Diagnosis Related Group system.

## Background

In many countries health care systems have developed tools and methodologies for the construction of quality of care indicators and efficiency of systems. The Agency for Health Research and Quality (AHRQ) constructed a series of indicators known as “Quality Indicators – Measuring Healthcare Quality”, which developed quality of care benchmarks among hospitals in the United States [1].

The results of these assessments have been systematically used to improve care quality and performance. For example, in April 2002 the UK Department of Health introduced a new system of financing hospitals, called “payment by results” [2]. The plan aimed to withdraw funds from providers who did not deliver adequate levels of elective care, and give additional funds to those providers delivering higher-quality care. Another example is the “Best Practice Tariffs” (BPT) [3], which was introduced by the UK National Health Service to improve the quality of care in specific areas on the basis of selected performance indicators. For patients with hip fracture, care must be quickly and carefully organized to ensure that the most positive outcomes are achieved. In this specific case the BPT consists of a base tariff, with an additional payment if the six clinical characteristics of best practice reported through the National Hip Fracture Database have been met.

Since 2002, outcome research programs (“The Mattoni-Outcome Project” and the “Progr.Es.Si. Project”) in Italy have been conducted on behalf of the Ministry of Health at the national level [4]. Many Italian regions have promoted the improvement of quality in health care through the modulation of hospital tariffs thus discouraging inappropriate care. As a result, we have observed a marked reduction in hospital admissions in favor of an increase in day care admissions.

In particular, the Regional Health Care Evaluation Program (P.Re.Val.E.) [5,6] elaborated and publicly released 54 hospital care outcome and process indicators in the Lazio region. Among the indicators measuring quality in the orthopedic specialty specific interest was addressed to hip fracture. These indicators take into account elderly patients admitted for hip fractures with regards to mortality within 30 days, waiting time to surgery, and proportion of interventions within 48 h of admission. This program emphasized critical issues in hospital treatment of elderly patients with hip fracture, highlighting performances far below the standard of appropriateness in the Lazio region.

Since 1992 in Italy, hospital reimbursement has been based on a prospective payment system known as the Diagnosis Related Group (DRG) system. The National Health Service pays hospitals a flat rate per case for inpatient hospital care so that hospitals are rewarded for efficiency or are stimulated to become more efficient. Since the establishment of remuneration based on the DRG system, tariff modulation mechanisms were introduced with the aim of reducing inappropriate care. In 2009 a regional act [7] determined the adoption of a clinical pathway for elderly patients with hip fracture by Lazio hospitals, and introduced a compensation system based on quality of health care, as in a pay-for-performance model [8]. The DRG reimbursement rate for Lazio providers was linked to hospital performance. In fact, since 2010 the full DRG rate has been paid only for patients that have undergone surgical treatment within 48 h of admission, while rates for surgeries performed more than 48 h after admission have been proportionally reduced on the basis of the interval between admission and surgery [7].

The pay-for-performance system only targeted hospitals that are really paid by the DRG system. During the study period, not all Lazio hospitals were fully paid on the basis of DRG: “Local health unit hospitals” actually have a fixed amount of money for health care spending, as in a global budgeting system, independent of DRG; “Public hospital corporations and teaching hospitals” have a global budget for almost all health services and are partially reimbursed by the DRG system; “Classified hospitals” (hospitals owned by religious congregations) are paid by the DRG system for almost all health care services, and have a budget to cover, at least in part, the remaining heath care spending; “Private hospitals” are entirely paid by the DRG system.

The objective of the present study was to compare the proportion of surgeries for hip fracture performed within 48 h of admission among Lazio hospitals according to different payment systems, before and after the implementation of the regional act.

## Methods

### Data sources

This study is based on information from the Hospital Information System (HIS) and the Healthcare Emergency Information System (HEIS). Discharge abstracts for all hospitals are routinely collected by the HIS and contain: patient demographic data (gender, age), admission and discharge dates, up to 6 discharge diagnoses (International Classification of Disease, 9th Revision, Clinical Modification [ICD-9-CM]), medical procedures or surgical interventions (up to 6), and status at discharge (alive, dead, transferred to another hospital). The HEIS collects all Emergency Department (ED) visit records in the Lazio region, including information on patients’ characteristics, four categories of severity based on triage (red, yellow, green, and white), main diseases, some clinical parameters, performed treatments, and diagnoses at discharge (most information is codified using ICD-9-CM codes).

### Study population

We conducted a retrospective cohort study of patients aged 65 years and older who were residing in the Lazio region and admitted to an acute care hospital for a hip fracture (ICD-9-CM diagnosis codes 820.0–820.9 in any position) between the period preceding the pay performance act (1 July 2008 to 30 June 2009, Period 1) and the period after the pay performance act was enacted (1 July 2010 to 30 June 2011, Period 2). All hospitalizations for hip fracture occurring between 1 July 2009 and 30 June 2010 were excluded from the analysis as it was considered as a transition period during which the regional act was developed based on quality of health care.

We excluded admissions of patients according to the following criteria:

– hospitalized for hip fracture in the previous 2 years;

– transferred from another acute care hospital or ED (patients admitted to a given ED or hospital for hip fracture and coded as 'transferred from’ another unidentified acute care facility or ED);

– had multiple significant traumas (DRGs 484–487);

– had a principal or secondary diagnosis of malignant neoplasm (codes 140.0–208.9) at the index admission (current admission for hip fracture) or at previous hospitalizations during the last 2 years.

– directly admitted to an intensive care unit;

– died within 48 h of admission without intervention (patients who could not have undergone surgery owing to poor baseline clinical conditions);

– admissions not remunerated by the National Health Service (e.g., admission remunerated by private insurance or by an out-of-pocket fee);

– admissions to hospitals with a volume of hip surgery (hip fracture) of less than 50 per year during Period 2.

### Outcome

The outcome under study is the surgery within 48 h (0–1 day) of hospital arrival. The date of hospital arrival corresponded to the date of the index or ED admission. The interventions were identified by the following ICD-9-CM codes: total or partial hip replacement (codes 81.51, 81.52) and reduction of fracture (codes 79.00, 79.05, 79.10, 79.15, 79.20, 79.25, 79.30, 79.35, 79.40, 79.45, 79.50, 79.55).

### Exposure: hospital payment types

Hospitals were classified according to hospital payment type:

1. Local health unit hospitals

2. Public hospital corporations and teaching hospitals

3. Religious hospitals

4. Private hospitals

### Comorbidities

Risk factors potentially associated with the outcome under study were chosen among the conditions identified in the literature [9-11]. Comorbidities were identified on the basis of ICD-9-CM codes registered either at the index hospitalization or at previous hospital or ED admissions during the last 2 years [12,13]. Acute events that occurred during the index admission that could be considered as complications of care were not included. Details and ICD-9-CM codes are reported in the Additional file 1.

### Statistical analysis

The proportions of surgery performed within 48 h of hospital arrival were calculated. We used multivariate regression analysis to assess the effect of hospital payment type on likelihood of surgery performed within 48 h of hospital arrival, adjusting for other factors (age, gender and comorbidities) that could affect the outcome under study. Among all factors potentially associated with the outcome under study, age and gender were considered a priori risk factors; the others were selected using a stepwise bootstrap procedure to assign an importance rank of predictors in the logistic regression analysis. In this approach, the logistic regression with all predictors is run 100 times on random samples drawn with replacement from the original data set. Only the risk factors identified at least 30 times with a significance level of 5% were included in the predictive model.

To estimate the adjusted proportion of surgery within 48 h of hospital arrival, a multivariate logistic regression analysis with no intercept, including centered covariates and an interaction term between types of hospital payment and study period, was applied. This model estimates log odds of surgery within 48 h with respect to hospital payment type allowing comparisons between types of hospital payment before and after the regional act.

Adjusted proportions were obtained for each level of hospital payment type by back-transforming parameter estimates with the following formula [14]:

$$\text{Adj}\;\text{proportion}=\left[\text{exp}\left(\text{estimate}\right)/\left(1+\text{exp}\left(\text{estimate}\right)\right)\right]*k$$

where k is a correction coefficient, introduced to take into account the nonlinear nature of the logistic model.

K is calculated as follows:

$$K=\text{actual}\;\text{number}\;\text{of}\;\text{events}/\sum_{j=1}^{m}p_{j}*n_{j}$$

where *p*_*j*_ are the adjusted proportions, *n*_*j*_ is the group size, and *m* is the number of groups.

Relative risks by hospital payment type were estimated to compare the proportion of surgeries within 48 h between Period 1 and Period 2.

All analyses were undertaken using SAS Version 9.2.

The data used for the study are not openly available. The Department of Epidemiology has been authorised by the Regional Health Authority to use the data.

## Results

The main characteristics of the study population included in each period are shown in Table 1. We studied a total of 12,433 admissions for hip fracture in the Lazio region: 6,043 during the period preceding the pay-for-performance act (Period 1) and 6,390 during the period after pay-for-performance act (Period 2). The mean age was 82.7 (SD: 7.2) years in Period 1 and 83.2 (SD: 7.1) years in Period 2. The proportion of females varied from 77.2% to 81.1%. Patient characteristics, including distribution by hospital payment type, were similar during both periods (Table 1). The crude proportion of interventions performed within 48 h was higher during Period 2 than Period 1 (22.2% and 11.7%, respectively). The hospital’s characteristics before and after the regional act, per types of hospital payment, are shown in Table 2.

**Table 1:** Characteristic of patients admitted with diagnosis of hip fracture in Lazio, before and after the pay performance act

**Patients characteristics**	**Before pay performance act (period 1)**	**After pay performance act (period 2)**
	**n = 6,043**	**n = 6,390**
Age (mean, SD)	82.7 (7.2)	83.2 (7.1)
	**%**	**%**
Gender		
Female	77.2	81.1
Obesity (in the previous 2 years)	0.4	0.4
Obesity (current admission)	0.4	0.5
Hemiplegia and other paralytic syndromes (in the previous 2 years)	0.5	0.3
Hemiplegia and other paralytic syndromes (current admission)	0.4	0.3
Other forms of chronic ischemic heart disease (in the previous 2 years)	9.9	9.3
Heart failure (in the previous 2 years)	4.9	5.7
Vascular disease (in the previous 2 years)	2.7	2.5
Vascular disease (current admission)	0.6	0.9
Cerebrovascular disease (in the previous 2 years)	9.0	8.4
Cerebrovascular disease (current admission)	3.1	3.1
Diabetes (in the previous 2 years)	6.2	6.2
Blood disorders (in the previous 2 years)	4.6	5.0
Hypertension (in the previous 2 years)	14.4	13.2
COPD (in the previous 2 years)	5.8	4.9
Chronic renal disease (in the previous 2 years)	3.3	3.5
Chronic renal disease (current admission)	3.2	4.2
Types of hospital payment		
Local health unit	54.6	55.9
Public and teaching	23.2	23.2
Classified	8.4	9.8
Private	13.9	11.1

**Table 2:** Characteristic of hospitals per types of hospital payment, before and after the pay performance act

**Types of hospital payment**	**Before pay performance act**				**After pay performance act**			
	**(Period 1)**				**(Period 2)**			
	**n = 6,043**				**n = 6,390**			
	**N. hospitals**	**Hospital admissions for a hip fracture**			**N. hospitals**	**Hospital admissions for a hip fracture**		
		**N**	**Median**	**Interquartile range (IQR)**		**N**	**Median**	**Interquartile range (IQR)**
**Local health unit**	23	3298	1650	1649	23	3571	1786	1786
**Public and teaching**	7	1402	702	701	7	1484	743	742
**Classified**	4	505	253	252	4	624	313	312
**Private**	7	838	420	419	7	711	356	356

The risk factors included in the predictive model are shown in the Additional file 2. All comorbidities included in the predictive model reduced the likelihood of hip fracture surgery within 48 h. In contrast, the likelihood increased with age and in female patients. Differences between crude and adjusted OR were not observed.

The results of the association between hospital payment type and hip fracture surgery within 48 h are reported in Table 3. We observed a lower probability of undergoing surgery within 48 h during Period 1 (range 8.9%-18.3%) than during Period 2 (range 19.8%-26.0%), for all hospital payment types. We observed a higher probability of hip fracture surgery within 48 h during Period 2 than during Period 1 by hospital payment type (local health unit: adjusted RR = 2.07, p < 0.001; public and teaching hospitals: adjusted RR = 1.42, p < 0.001; classified hospitals: adjusted RR = 2.00, p < 0.001; private hospitals: adjusted RR = 2.80, p < 0.001). Finally, differences between crude and adjusted proportion of patients receiving surgery within 48 h were not observed.

**Table 3:** Association between types of hospital payment and surgery within 48 hours

**Types of hospital payment**	**Before pay performance act**				**After pay performance act**				**Comparison between period**		
	**(Period 1)**				**(Period 2)**						
	**Hip surgery**				**Hip surgery**				**Hip surgery**		
	**n**	**Proportion**	**Proportion within 48 h**	**Adjusted proportion within 48 h**	**n**	**Proportion**	**Proportion within 48 h**	**Adjusted proportion within 48 h**	**Percentage change proportion within 48 h**	**Adjusted RR**	**p value**
**Local health unit**	3298	86.0	9.6	9.6	3571	86.0	19.8	19.7	51.7	2.07	0.000
**Public and teaching**	1402	94.3	18.3	18.4	1484	94.1	26.0	26.2	29.8	1.42	0.000
**Religious**	505	90.9	12.1	11.9	624	93.9	23.7	23.8	49.1	2.00	0.000
**Private**	838	86.2	8.9	9.0	711	88.9	25.2	25.2	64.5	2.80	0.000
**TOTAL**	6043				6390						

## Discussion

An improvement in the proportion of patients receiving early surgery for hip fracture after the introduction of the pay-for-performance act was observed in cases remunerated through all hospital payment types. However, the largest increase of surgery within 48 h was observed for private hospitals. This improvement resulted in a strong reduction of the diversity of the interval from admission to surgery across hospital types. In the period preceding the introduction of the pay-for-performance act, the patients admitted to private hospitals had the lowest likelihood of receiving surgical intervention for hip fracture within 48 h, while less than one-half of the likelihood was observed in public and teaching hospitals. During the period after the introduction of the pay-for-performance act, public hospitals still exhibited the greatest proportion of patients receiving hip surgery within 48 h; however, the greatest increase was observed in local health unit and private hospitals.

In Italy, the National Health System provides equal access to healthcare for all citizens and residents through a mixture of public and private services. In the Lazio region (5.7 million inhabitants and healthcare budget of 1.1 million euros/year) there are 41 private and public hospitals that perform hip surgery (Table 2). The hospitals are very different in size, and serve areas that differ for age composition and distribution of diseases [15].

Our results seem to confirm the hypothesis that the introduction of a compensation system based on quality of health care, such as the pay-for-performance model, is associated with improved quality of care for elderly patients with hip fracture. This finding is especially true for hospitals entirely paid by the DRG system, as shown in a study that compared the quality improvement programs in Lazio and Toscana [16].

The impact of pay-for-performance in the Lazio region can be related to the fact that the hospitals had plenty of room for improvement from baseline [17], given the low proportion of patients receiving surgery within 48 hours [16].

Even if the evidence of the pay-for-performance model effectiveness remains weak [18], its implementation was a necessary measure to improve the performance of the health care systems in Lazio and in other Italian regions. The compensation system of a pay-for-performance needs to be linked with important clinical outcomes [19-21], in Lazio hospital remuneration was related to the proportion of hip surgery performed within 48 hours. The Lazio regional act was aimed at implementing the pay-for-performance model uniformly to all providers in the region so that its effects were not diluted [16], however it is noteworthy that the pay-for-performance model could have an impact at the hospital level rather than at the individual or team level [17,19,22]. Furthermore, healthcare providers could have affected the impact of pay-for-performance program as different ownership types might have implemented different incentives related to the uptake of the pay-for-performance model [23].

The increase of interventions performed within 48 h of admission may be due to the development of programs for public release. In 2007, P.Re.Val.E [5,6] was conducted with the aim of improving the quality of health care; in 2008, the Agency for Public Health of Lazio designed a clinical pathway for elderly patients with hip fracture. The impact of these programs on the quality of health care for orthopedic patients compared with other Italian regions was evaluated in a recent study [16]. Overall, there is no clear evidence regarding an association between public reporting and improved quality of care, even though some studies suggest that public reporting may motivate quality improvement activities [24,25]. A previous study found little evidence of an association between the introduction of result-based payment and a change in the quality of care. In this study, Farrar et al. used three variables to measure quality: in-hospital mortality, 30-day postsurgical mortality, and emergency readmission after treatment for hip fracture [26]. Finally, the improvement of quality of health care for elderly patients admitted for hip fracture may be associated with the “announcement effect” of the regulatory act on the Lazio providers.

There are other differences among Lazio hospitals that could affect the capacity of reacting to the changing payment methods. The differences in the management of elderly patients with hip fracture in hospitals depends on the different distribution of elective or emergency patients and the relative waiting time to surgery and on the clinical practices of the hospital specialties. The distribution of socio-economic characteristics of elderly patients with hip fracture could be different among Lazio hospitals and there is evidence that more disadvantaged people could experience longer waiting times [27]. However, the socioeconomic inequalities in the waiting times for surgery were reduced in Lazio region after the implementation of P.Re.Val.E. [28].

The strengths of this study include the before-after design, the large data sample available for analysis, the validated algorithm for cohort selection and variable definitions, and the robust outcome. However, the limitations should also be considered. The study relies entirely on administrative data and, despite the broad and valued use of administrative data for health care research, hospital discharge data have several limitations that have been recognized repeatedly [29]. In addition, although several covariates were included in the models to adjust for differences in patient characteristics, unmeasurable or unmeasured covariates that might affect the likelihood of intervention within 48 h of admission may not have been taken into account. However, the lack of major differences between the crude and adjusted proportion of patients receiving surgery within 48 h and the homogeneity of results by hospital payment type suggest that these covariates may not affect results. Furthermore, different coding practices across hospitals and misclassification of comorbidity are unlikely to be associated with hospital payment type.

## Conclusion

In conclusion, this study contributes to the debate about the impact of performance-based programs on quality of care and the efficiency of systems.

## Competing interests

The authors declare that they have no competing interests.

## Authors’ contributions

CP: study conception and design, data analysis, interpretation of data. PL: study conception and design, interpretation of data. FD: study design, data analysis, interpretation of data. PCA: study conception and design, interpretation of data. BM: study conception and design, interpretation of data. DM: study conception and design, interpretation of data. All authors read and approved the final manuscript.

## Pre-publication history

The pre-publication history for this paper can be accessed here:

http://www.biomedcentral.com/1472-6963/13/393/prepub

## Supplementary Material

Additional file 1 List of comorbidities used for risk adjustment. Click here for file

Additional file 2 Comorbidities included in a model to predict surgery within 48 hours. Click here for file

## Data used for the study

The data used for the study are not openly available. The Department of Epidemiology has been authorised by the Regional Health Authority to use the data.
