# Supplementary material for: The impact of a pay-for-performance system on timing to hip fracture surgery: experience from the Lazio Region (Italy)
Source: BMC Health Serv Res. 2013 Oct 7;13:393. doi: 10.1186/1472-6963-13-393 (PMC3852766; doi:10.1186/1472-6963-13-393)
Supplement: Additional file 1 — List of comorbidities used for risk adjustment. [file 1472-6963-13-393-S1.doc]

**Additional file 1: List of comorbidities used for risk adjustment**

| **Risk factor** | **ICD-9-CM code** | |
| --- | --- | --- |
|  | **Current admission** | **Previous hospital or ED admissions** |
| Diabetes |  | 250.1-250.9 |
| Nutritional deficiencies | 260-263, 783.2, 799.4 | 260-263, 783.2, 799.4 |
| Obesity | 278.0 | 278.0 |
| Blood disorders | 280-285, 288, 289 | 280-285, 288, 289 |
| Dementias including Alzheimer’s disease | 290.0-290.4, 294.1, 331.0 | 290.0-290.4, 294.1, 331.0 |
| Parkinson’s disease | 332 | 332 |
| Hemiplegia and other paralytic syndromes | 342, 344 | 342, 344 |
| Hypertension |  | 401-405 |
| Previous myocardial infarction | 412 | 410, 412 |
| Other forms of chronic ischemic heart disease |  | 411, 413, 414 |
| Heart failure |  | 428 |
| Ill-defined descriptions and complications of heart disease |  | 429 |
| Rheumatic heart disease | 393-398 | 391, 393-398 |
| Cardiomyopathy | 425 | 425 |
| Acute endocarditis and myocarditis |  | 421, 422 |
| Other heart conditions | 745, V15.1, V42.2, V43.2, V43.3, V45.0 | 745, V15.1, V42.2, V43.2, V43.3, V45.0 |
| Cardiac arrhythmias |  | 426, 427 |
| Cerebrovascular disease | 433, 437, 438 | 430-432, 433, 434, 436, 437, 438 |
| Vascular disease | 440-448 (excluding 441.1, 441.3, 441.5, 441.6, 444) , 557.1 | 440-448, 557 |
| COPD |  | 491-492, 494, 496 |
| Chronic renal disease | 582-583, 585-588 | 582-583, 585-588 |
| Other chronic disease (liver, pancreas, intestine) | 571-572, 577.1-577.9, 555, 556 | 571-572, 577.1-577.9, 555, 556 |
| Rheumatoid arthritis and other inflammatory polyarthropathies | 714 | 714 |
| Osteoporosis and other disorders of bone and cartilage | 733.0 | 733.0 |
