# Supplementary material for: The impact of a pay-for-performance system on timing to hip fracture surgery: experience from the Lazio Region (Italy)
Source: BMC Health Serv Res. 2013 Oct 7;13:393. doi: 10.1186/1472-6963-13-393 (PMC3852766; doi:10.1186/1472-6963-13-393)
Supplement: Additional file 2 — Comorbidities included in a model to predict surgery within 48 hours. [file 1472-6963-13-393-S2.doc]

**Additional file 2 – Comorbidities included in a model to predict surgery** within 48 hours.

| **Risk factors** | **n** | **Crude Odds Ratio** | **Adjusted Odds Ratio** | **p adj** |
| --- | --- | --- | --- | --- |
| Age (year) | - | 1.01 | 1.01 | 0.024 |
| Gender (F vs M) | 9563 | 1.20 | 1.16 | 0.014 |
| Obesity | 46 | 0.87 | 0.97 | 0.946 |
| Obesity (current admission) | 53 | 1.92 | 1.87 | 0.042 |
| Hemiplegia and other paralytic syndromes | 48 | 0.10 | 0.12 | 0.039 |
| Other forms of chronic ischemic heart disease | 1161 | 0.69 | 0.77 | 0.006 |
| Heart failure | 640 | 0.60 | 0.68 | 0.003 |
| Vascular disease | 314 | 0.43 | 0.50 | 0.001 |
| Cerebrovascular disease | 1054 | 0.84 | 1.01 | 0.950 |
| Cerebrovascular disease (current admission) | 375 | 0.65 | 0.68 | 0.018 |
